# *RAD52* Variants Predict Platinum Resistance and Prognosis of Cervical Cancer

**DOI:** 10.1371/journal.pone.0050461

**Published:** 2012-11-29

**Authors:** Ting-Yan Shi, Gong Yang, Xiao-Yu Tu, Jing-Min Yang, Ji Qian, Xiao-Hua Wu, Xiao-Yan Zhou, Xi Cheng, Qingyi Wei

**Affiliations:** 1 Cancer Research Laboratory, Fudan University Shanghai Cancer Center, Department of Oncology, Shanghai Medical College, Fudan University, Shanghai, China; 2 Department of Pathology, Fudan University Shanghai Cancer Center, Department of Oncology, Shanghai Medical College, Fudan University, Shanghai, China; 3 Department of Gynecologic Oncology, Fudan University Shanghai Cancer Center, Department of Oncology, Shanghai Medical College, Fudan University, Shanghai, China; 4 State Key Laboratory of Genetic Engineering, The Institute of Genetics, School of Life Sciences, Fudan University, Shanghai, China; 5 Department of Epidemiology, The University of Texas M. D. Anderson Cancer Center, Houston, Texas, United States of America; 6 National Population and Family Planning Key Laboratory of Contraceptive Drugs and Devices, Shanghai Institute of Planned Parenthood Research, Shanghai, China; Baylor College of Medicine, United States of America

## Abstract

*RAD52* is an important but not well characterized homologous recombination repair gene that can bind to single-stranded DNA ends and mediate the DNA-DNA interaction necessary for the annealing of complementary DNA strands. To evaluate the role of *RAD52* variants in the response of tumor cells to platinum agents, we investigated their associations with platinum resistance and prognosis in cervical cancer patients. We enrolled 154 patients with cervical squamous cell carcinoma, who had radical surgery between 2008 and 2009, and genotyped three potentially functional *RAD52* variants by the SNaPshot assay. We tested *in vitro* platinum resistance and RAD52 expression by using the MTT and immunohistochemistry methods, respectively. In 144 cases who had genotyping data, we found that both the rs1051669 variant and RAD52 protein expression were significantly associated with carboplatin resistance (*P* = 0.024 and 0.028, respectively) and rs10774474 with nedaplatin resistance (*P* = 0.018). The rs1051669 variant was significantly associated with RAD52 protein expression (adjusted OR = 4.7, 95% CI = 1.4−16.1, *P* = 0.013). When these three *RAD52* variants were combined, progression-free survival was lower in patients who carried at least one (≥1) variant allele compared to those without any of the variant alleles (*P* = 0.047). Therefore, both *RAD52* variants and protein expression can predict platinum resistance, and *RAD52* variants appeared to predict prognosis in cervical cancer patients. Large studies are warranted to validate these findings.

## Introduction

Cervical cancer is the third most commonly diagnosed cancer and the fourth leading cause of cancer death in women, accounting for 9% (529,800) of all new cancer cases and 8% (275,100) of all cancer deaths among women in 2008 in the world [1]. More than 85% of these cases and deaths occur in the developing countries, including China [1]. Concurrent chemoradiation therapy is the standard therapy used most often for patients with cervical squamous cell carcinoma (CSCC) who have locally advanced [2], [3] and recurrent and/or metastatic [4] diseases. However, primary or acquired chemoresistance is a serious clinical problem that contributes to disease recurrence, progression and disease-specific mortality [5]–[7]. The mechanism underlying heterogenous response of the patients is multifactorial and can include multiple genetic factors. Some of these genetic factors can reliably and prospectively be assessed for their role in determining response to anticancer agents.

DNA repair is mainly responsible for repairing and/or removing platinum-DNA adducts. This DNA repair involves the coordinated activities of more than 20 enzymes that remove and restore a segment of DNA containing a bulky adduct [8]. Homologous recombination repair (HRR) is a major DNA repair mechanism to repair double-strand breaks (DSBs) during the S and G2 phases [9], which is a cellular defense mechanism against cytotoxic effects of platinum-based chemotherapeutic agents [10]–[13]. *RAD52*, as a relatively less well-characterized HRR gene, spans 37.6 kb on chromosome 12p12.2-13 and codes for a protein with 417 amino acids, which can bind to single-stranded DNA ends and mediate the DNA-DNA interaction necessary for the annealing of complementary DNA strands [14]. Recently, Tassone et al. reported that an overexpression of *RAD52* mRNAs in BRCA1-defective HCC1937 cells was associated with high sensitivity to platinum-derived compounds [15]. Therefore, we hypothesize that *RAD52* may be involved in platinum resistance in cancer cells.

Single nucleotide polymorphisms (SNPs) in genes involved in DNA repair may affect protein binding sites or affinity or cause changes in protein structure and thus may modify gene functions and render cancer cells more sensitive to platinum treatment [16], [17]. To date, few reported studies have investigated associations of *RAD52* SNPs with the risk of malignancy [18], [19], and none have assessed this with chemoresistance. In the present study, we evaluated the association of three potentially functional *RAD52* SNPs with *in vitro* platinum resistance and prognosis in patients with CSCC.

## Materials and Methods

### Ethics Statement

This project was approved by the Institutional Review Board of Fudan University Shanghai Cancer Center (FUSCC). A written informed consent was obtained from all recruited individuals, and each clinical investigation was conducted according to the principles expressed in the Declaration of Helsinki consent.

### Patients

In the present study, we recruited 154 CSCC patients who had radical hysterectomy and pelvic lymphadenectomy between March 2008 and March 2009 at the Fudan University Shanghai Cancer Center (FUSCC). All cases were histologically confirmed to be squamous cell carcinoma by two gynecologic pathologists (XYT and GY). The detailed clinical information was extracted from the patients’ electronic database at FUSCC and included age, tumor stage (FIGO, International Federation of Gynecology and Obstetrics, 2009), tumor size (i.e., the largest tumor diameter reported by the pathologist after surgical resection), pelvic lymph node (LN) metastases, lympho-vascular space invasion (LVSI) and depth of cervical stroma invasion. Patients were followed every three months for the first two years, every six months for the next two years, and annually for the following years thereafter. The analysis of all blood and tissue samples was carried out in a blinded fashion with regard to patients’ chemoresistance and survival status.

### SNP Selection

The SNPs were selected from the NCBI dbSNP database (http://www.ncbi.nlm.nih.gov/projects/SNP), the International HapMap Project database (http://hapmap.ncbi.nlm.nih.gov/) and the SNP function prediction (FuncPred) software (http://snpinfo.niehs.nih.gov/snpfunc.htm) based on four criteria: 1) located at the two ends of the *RAD52* gene [i.e., 5′-flanking, 5′-untranslated region (UTR), 3′-UTR, 3′-flanking], 2) had a minor allele frequency of at least 5% in Chinese populations, 3) in low linkage disequilibrium by using an *r* ^2^ threshold of 0.8 for each other, and 4) predicted as potentially functional SNPs at microRNA (miRNA) binding sites or transcription factor binding sites. As a result, three *RAD52* SNPs were selected, and they are rs1051669G>A (3′-UTR), rs10774474A>T (5′-flanking) and rs11571378T>A (5′-flanking).

### DNA Extraction and Genotyping

Genomic DNA was obtained from whole blood samples using a QuickGene DNA Whole Blood Kit (Fujifilm Co., Tokyo, Japan) according to the manufacturer’s instructions. DNA purity and concentration were determined by spectrophotometric measurement of absorbance at 260 and 280 nm by Synergy™ 4 Multi-Mode Microplate Reader (BioTek, Winooski, VT).

The selected SNPs were genotyped using the multiplex SNaPshot assay. The primers for PCR amplification and SNaPshot extension were designed to have an annealing temperature around 60°C using Primer5 software. To test for possible repetitive sequences, primers were aligned with the GeneBank database using the BLAST online tool. AutoDimer Software was used in the detection of potential hairpin structures and possible primer-dimer combinations. All primers were synthesized by Sangon Biotech Co., Ltd. (Shanghai, China). After amplification and purification, the PCR products were mixed and used as the template in the SNaPshot extension reaction. Then, electrophoresis was performed on the ABI 3130 Genetic Analyzer (Applied Biosystems, Foster City, CA) to check the quality of PCR products. The genotyping data was finally analyzed by Peak Scanner Software version 1.0 (Applied Biosystems). Ten percent of the samples were randomly selected to be sequenced. As a result, the mean genotyping rate was 93.5% by using the multiplex SNaPshot assay. The discrepancy rate in all positive controls (i.e., duplicated samples, overlapping samples from previous studies and samples randomly selected to be sequenced) was less than 0.1%.

### The MTT Assay [20]

In performing the MTT assay, we considered previously reported peak plasma concentrations (PPCs) for the four selected platinum agents: cisplatin 10 µg/ml (Qilu Pharmaceutical Co., China; clinical dosage 100 mg/m^2^) [21], carboplatin 20 µg/ml (Qilu Pharmaceutical Co., China; clinical dosage 450 mg/m^2^) [22], nedaplatin 10 µg/ml (Nanjing Dongjie Pharmaceutical Co., China; clinical dosage 100 mg/m^2^) [23] and oxaliplatin 12 µg/ml (Nanjing Pharmaceutical Factory Co., China; clinical dosage 130 mg/m^2^) [24]. In the actual assay, we used 10×PPC as the final working concentration for each of these platinum agents as recommended previously [25], [26].

Histopathologically confirmed fresh tumor tissues obtained at surgery were cut into pieces of smaller than 5×5 mm and suspended in the RPMI 1640 medium (Sigma–Aldrich Co., St. Louis, MO) supplied with 15% fetal bovine serum (Gibco, Langley, OK) at room temperature. Suspended cancer cells were poured over a 150 µm sterile steel mesh placed in the dish. Percoll discontinuous gradient centrifugation with 400 g was used to separate and purify cancer cells as recommended previously [20]. Cancer cells were identified by using the H&E stained morphological examination and then resuspended at a concentration of 1×10^5^ viable cells/ml and cultured in 96-well microplates at 37°C in a 5% CO_2_ incubator. On day two, 90 µl RPMI 1640 medium supplied with 15% fetal bovine serum and 10 µl of the solutions with 100×PPC for each of the selected agents were mixed for 48 h. For each patient, 100 µl of cancer cell suspension without platinum agents were cultured as negative assay controls. On day four, 20 µl MTT (5 mg/mL, Shanghai Lanji Co., China) was added into each well at 37°C for 4 h, and then 100µl solution buffer (10% SDS, 5% isobutanol and 0.012 mol/L HCl) were added into each well overnight to dissolve formazan crystals. Optical densities (OD_570 nm_) were measured by Microplate Reader (BIO-RAD550, Hercules, CA). The inhibition rate was obtained by using the formula of (1−OD_platinum_/OD_control_)×100%.

### Tissue Microarray, Immunohistochemistry (IHC) Assay and the Assessment of Immune Staining

The portions of tumor/normal tissue to be used for the tissue microarray were selected from a representative tumor/normal area in the corresponding H&E stained section of each block by two gynecologic pathologists (XYT and GY). A 10×12 (120 cores) array was made by the Tissue Bank of FUSCC, which also included 17 controls of normal cervical tissues. For each patient, two cores were made from separate sources. IHC was performed on 5 µm-thick tissue sections prepared from formalin-fixed, paraffin-embedded tissue from the constructed tissue microarray block using antibody against RAD52 [sc-8350, rabbit polyclonal antibody, Santa Cruz Biotechnology (Inc., Santa Cruz, CA), 1∶50 dilution] and ChemMate™ EnVision™ detection kit (DAKO, Glostrup, Denmark). A known positive sample was included as a positive control. For the negative control, the primary antibody was replaced with nonimmune rabbit serum.

IHC staining results were independently scored by two gynecologic pathologists (XYT and GY), who were blinded to patient information, using BX51 microscope and DP25 cameras (Olympus Co., Tokyo, Japan). The scoring system was based on both the percentage of positive cells and staining intensity, as described previously [27]. The assessment of the protein expression status was defined as negative (≤2+) and positive (>2+). For cores that were uninterpretable because of tissue loss or lack of cells, a score of not applicable (N/A) was given.

### Statistical Methods

Because the platinum-inhibition rates did not follow normal distributions, we performed the nonparametric *Wilcoxon* test and *Kruskal-Wallis* test to compare platinum-inhibition rates for each agent both between the agents and among different groups. Pearson’s χ^2^-test and logistic regression analysis were also used to evaluate the association between *RAD52* genotypes and protein expression. For the survival analysis, progression-free survival (PFS) and overall survival (OS) times were calculated from the date of first surgery to the date of disease recurrence and death, respectively. Patients without progression, lost to follow-up or died from other causes were censored at their last date of record. *Kaplan-Meier* survival estimate and log-rank test were calculated to evaluate PFS and OS. We performed Cox proportional hazards regression analysis to evaluate the effects of *RAD52* genotypes on the cumulative probability of survival in CSCC patients. Each reported *P* value was two-sided, and *P*<0.05 was used to infer the statistical significance. All analyses were performed using SAS software (version 9.1; SAS Institute, Cary, NC).

## Results

### Patient Characteristics and in vitro Platinum-inhibition Rates by the MTT Assay

Genotyping was unsuccessful in 10 cases after repeated assays. Therefore, the final analysis included 144 CSCC patients, whose clinical and pathological characteristics are summarized in **Table 1**. The patients’ median age at diagnosis was 46.5 years (range, 20–70 years). FIGO stage distribution was 68 (47.6%) stage IB, 67 (46.9%) stage IIA and eight (5.6%) stage IIB. Patients with a tumor size greater than 4 cm accounted for 43.8% (63/144) of the cases. Forty nine (34.0%) and 50 (34.7%) patients had positive pelvic LN metastases and positive LVSI, respectively. There were 109 (75.7%) cases whose cancer cells invaded more than 1/2 stroma layer of the cervix. The median follow-up was 37.6 months (range, 32.1–41.6 months), and there were 20 (13.9%) recurrences and 10 (6.9%) deaths during the follow-up period.

**Table 1:** Clinicopathologic characteristics of all patients with CSCC.

Characteristics	All subjects	
	N = 144	%
Age, yr, Mean (Range)	46.5 (20–70)	
FIGO Stage		
IB	68	47.6
IIA	67	46.9
IIB	8	5.6
Tumor size, cm		
≤4	81	56.3
>4	63	43.8
Pelvic LN		
Negative	95	66.0
Positive	49	34.0
LVSI		
Negative	94	65.3
Positive	50	34.7
Depth of cervical stroma invasion		
≤1/2	35	24.3
>1/2	109	75.7
Follow-up observation time, mo, Median (Range)	37.6 (32.1–41.6)	
Recurrence	20	13.9
Cancer-related death	10	6.9

CSCC, cervical squamous cell carcinoma; FIGO, International Federation of Gynecology and Obstetrics; LN, Lymph Node; LVSI, lympho-vascular space invasion.

**Figure 1:**
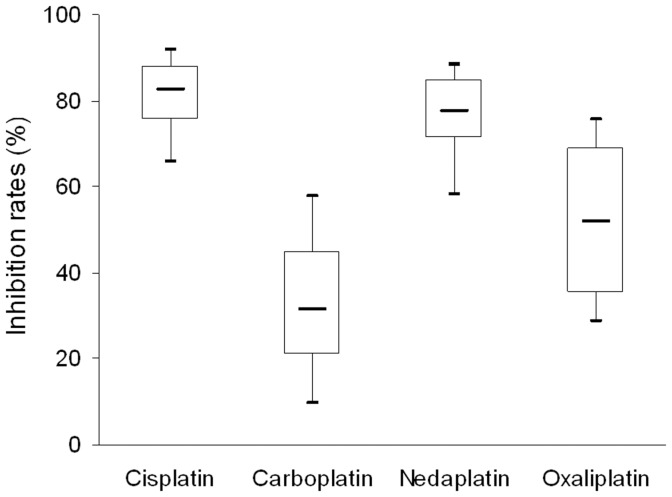
Boxplot for inhibition rates of four platinum agents in patients with cervical squamous cell carcinoma. The median *in vitro* inhibition rate of cancer cell growth by cisplatin, carboplatin, nedaplatin and oxaliplatin was 80.8%, 34.3%, 76.4% and 52.0%, respectively, and marked with “–”. Four groups showed a significant difference in inhibiting cancer cells (*Kruskal-Wallis* test, *P*<0.001). No significant difference in inhibition rates was observed between cisplatin and nedaplatin groups (*Wilcoxon* test, *P* = 0.269).

The median *in vitro* inhibition rate of cancer cell growth by cisplatin, carboplatin, nedaplatin and oxaliplatin was 80.8%, 34.3%, 76.4% and 52.0%, respectively (**Figure 1**). By using the *Kruskal-Wallis* test, we found that the four platinum agents showed a significant difference in inhibiting cancer cells (*Kruskal-Wallis* test, *P*<0.001; **Figure 1**). Cisplatin and nedaplatin both had a greater effect on inhibiting cancer cells than carboplatin did (*Kruskal-Wallis* test, *P*<0.001; **Figure 1**). Additionally, no significant difference in inhibition rates was observed between cisplatin and nedaplatin groups (*Wilcoxon* test, *P* = 0.269; **Figure 1**). For each platinum agent, we compared its inhibition rates in different groups according to various clinical and pathological characteristics, but no significant difference was found (data not shown).

### Association between RAD52 SNPs and in vitro Platinum-inhibition Rates

All the observed genotype distributions among the patients agreed with the Hardy-Weinberg equilibrium (HWE, *P* = 0.533 and 0.115 for rs1051669 and rs10774474, respectively), except for rs11571378 (*P* = 0.0004). Overall, the rs1051669A and rs10774474T variant alleles were significantly associated with platinum resistance. Cancer cells with rs1051669AA and rs10774474TT variant homozygotes had lower responses to carboplatin and nedaplatin, respectively (*Kruskal-Wallis* test, *P* = 0.024 and 0.018, respectively; **Table 2**). Specifically, by assuming a recessive genetic model, we found that patients who carried the rs10774474 TT genotype had a significantly increased resistance to nedaplatin, compared with those who carried AA/AT genotypes (*Wilcoxon* test, *P* = 0.027). The rs1051669 AA genotype carriers appeared to have increased carboplatin resistance compared with GG/AG genotype ones but this was not statistically significant (*Wilcoxon* test, *P* = 0.074). There was no significant association between the *in vitro* inhibition rates and the rs11571378 SNP.

**Table 2:** *RAD52* variants and protein expression levels as predictors of response to platinum agents in CSCC.

Variables	Cases N (%)	Median±SE (%)*P* a							
		Cisplatin		Carboplatin		Nedaplatin		Oxaliplatin	
rs1051669			0.804		**0.024**		0.917		0.576
GG	97 (67.4)	82.0±2.4		24.5±4.6		76.7±2.4		49.7±3.1	
AG	41 (28.5)	76.8±4.3		46.0±6.9		75.3±4.3		49.3±4.8	
AA	6 (4.2)	77.2±13.6		2.4±2.4		78.0±13.5		71.3±8.8	
rs10774474			0.432		0.813		**0.018**		0.127
AA	45 (31.3)	75.9±4.0		30.3±4.4		73.0±4.1		56.2±4.1	
AT	79 (54.9)	84.3±2.7		34.5±3.5		81.8±2.6		56.0±3.5	
TT	20 (13.9)	74.7±5.7		31.7±6.2		68.3±5.5		40.6±6.8	
rs11571378			0.204		0.188		0.384		0.083
TT	78 (54.2)	76.8±3.0		34.4±3.5		74.8±2.9		48.0±3.4	
AT	66 (45.8)	84.0±2.9		28.3±3.5		78.3±3.1		58.4±3.6	
RAD52 protein expression^b^			0.300		**0.028**		0.105		0.246
Negative	109 (78.4)	77.6±2.5		28.6±2.8		74.5±2.6		49.3±2.9	
Positive	30 (21.6)	84.2±3.8		50.1±5.7		81.4±3.5		52.3±5.3	

CSCC, cervical squamous cell carcinoma.

a *Kruskal-Wallis* test or *Wilcoxon* test were used to compare platinum-inhibition rates among three or two groups, respectively;

b Five cases were excluded because of tissue loss or lack of cancer cells.

The results were in **bold**, if *P*<0.05.

### Association between Clinicopathological Characteristics, RAD52 SNPs and Survival

**Figure 2:**
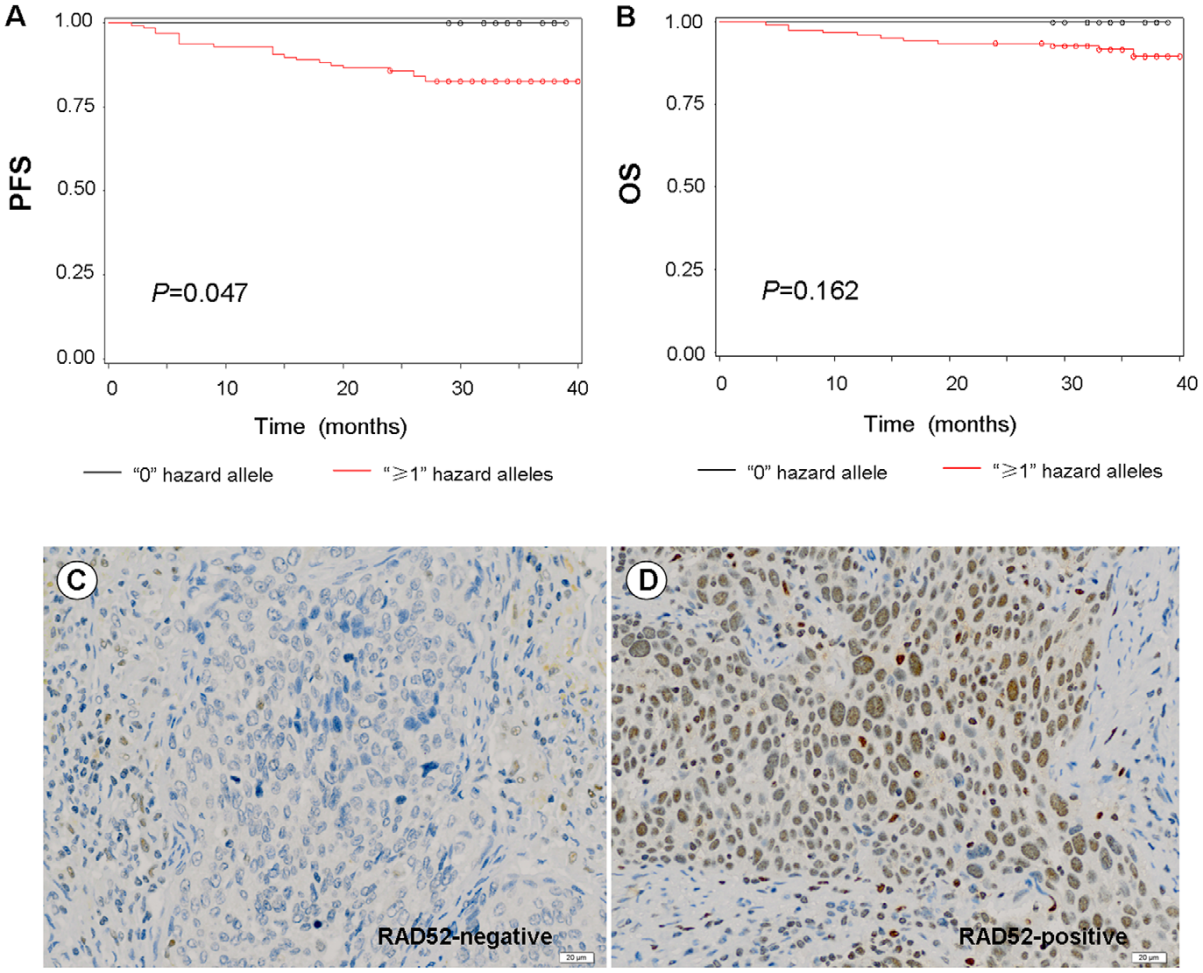
*Kaplan-Meier* survival estimates (A) progression-free survival and (B) overall survival by combined genotypes of the three selected *RAD52* SNPs. The patients carrying at least one (≥1) variant allele (i.e., rs10774474T, rs11571378A and rs1051669A) had poorer progression-free survival and overall survival compared to those without any of the variant alleles (log-rank test, *P* = 0.047 and 0.162, respectively). RAD52 (C) negative and (D) positive expression with low cytoplasmic background was detected by antibody sc-8350 (400×).

After adjusting for patients′ age, FIGO stage, tumor size, pelvic LN metastases, LVSI and depth of stromal invasion, we did not find an independent association of a single variant, alone, with patients′ survival (data not shown). However, when these three selected RAD52 SNPs were combined, patients carrying at least one (≥1) variant allele (i.e., rs10774474T, rs11571378A and rs1051669A) had poorer PFS and OS compared to those without any of the variant alleles (log-rank test, *P* = 0.047 and 0.162, respectively; **Figure 2A and 2B**).

### RAD52 SNPs Associated with RAD52 Protein Expression

To further evaluate biological plausibility underlying the observed association, we performed the IHC analysis of target tissues and found that RAD52 was localized to the nucleus (**Figure 2C and 2D**). The mean scores of RAD52 protein expression levels were 1.6±1.8 and 4.2±1.8 for cervical cancer and normal tissues, respectively. Of the 144 cases analyzed, 109 (75.7%) were RAD52 negative, 30 (20.8%) were RAD52 positive and five (3.5%) were RAD52 N/A. The positive expression rate (>2+) of RAD52 protein in cervical cancer tissues was 22% (30/139) compared to 88% in the normal tissues (15/17) (χ^2^-test, *P*<0.001). The genotype distributions of the rs1051669G>A, rs10774474A>T and rs11571378T>A SNPs in the RAD52-negative and positive patients are shown in **Table 3**. In the dominant genetic models, the rs1051669 variant AG/AA carriers showed a significantly decreased expression level of RAD52 protein in CSCC patients, compared with variant GG genotype carriers [logistic regression analysis, adjusted odds ratio (OR) = 4.7, 95% confidence interval (CI) = 1.4–16.1, *P* = 0.013; **Table 3**]. In addition, the RAD52-negative rate was significantly associated with poor response of cervical cancer cells to carboplatin (*Wilcoxon* test, *P* = 0.028; **Table 2**). However, we did not find any significant correlation between RAD52 protein expression and patients′ survival (data not shown).

**Table 3:** Logistic regression analysis of correlation between *RAD52* genotypes and protein expression in CSCC.

Variants Genotypes	RAD52 protein expression			*P* a	Crude OR(95% CI)	*P*	Adjusted OR(95% CI)^b^	*P* b
	Score (Mean±SD)	NegativeN (%)	PositiveN (%)					
All patients	1.6±1.8	109 (78.4)	30 (21.6)					
rs1051669				0.111				
GG	1.8±1.9	70 (73.7)	25 (26.3)		1.00		1.00	
AG	1.3±1.6	34 (87.2)	5 (12.8)		2.4 (0.9–6.9)	0.096	4.3 (1.3–14.8)	**0.020**
AA	1.2±1.1	5 (100)	0 (0)		–	0.976	–	0.980
AG/AA	1.3±1.5	39 (88.6)	5 (11.4)		2.8 (1.0–7.9)	0.053	4.7 (1.4–16.1)	**0.013**
Additive model					2.7 (1.0–7.3)	**0.043**	4.6 (1.4–15.0)	**0.013**
rs10774474				0.139				
AA	1.4±1.7	36 (83.7)	7 (16.3)		1.00		1.00	
AT	1.9±2.0	55 (72.4)	21 (27.6)		0.5 (0.2–1.3)	0.165	0.6 (0.2–1.8)	0.406
TT	1.2±1.4	18 (90.0)	2 (10.0)		1.8 (0.3–9.3)	0.512	1.6 (0.3–9.6)	0.589
AT/TT	1.7±1.9	73 (76.0)	23 (24.0)		0.6 (0.2–1.6)	0.312^d^	0.7 (0.3–2.0)	0.564^d^
Additive model					1.0 (0.5–1.9)	0.991	1.0 (0.5–2.1)	0.930
rs11571378				0.245				
TT	1.8±1.9	56 (74.7)	19 (25.3)		1.00		1.00	
AT	1.4±1.7	53 (82.8)	11 (17.2)		1.6 (0.7–3.8)	0.247	1.6 (0.7–3.7)	0.283

CSCC, cervical squamous cell carcinoma; OR, odds ratio; CI, confidence interval.

a χ^2^-test for genotype distributions between negative and positive RAD52 expression;

b Logistic regression models with the adjustment of age, FIGO stage, tumor size, pelvic LN, LVSI and depth of stroma invasion.

The results were in **bold**, if *P*<0.05.

## Discussion

Resistance to platinum-based chemotherapy is a challege in clinical practice [5]–[7]. In the present study, to evaluate platinum resistance, we performed the MTT assay that was widely used for chemical sensitivity test, and its overall accuracy was about 81.3% for predicting clinical effect in gynecological cancer [28]. We found that the inhibition rate of nedaplatin and cisplatin to cervical cancer cells are much higher than that of carboplatin and oxaliplatin, regardless of clinical and pathological variables. This finding is consistent with the current NCCN guideline recommendations that cisplatin be the first choice for the chemotherapy of cervical cancer. Our findings regarding nedaplatin are also consistent with published clinical data. Increasing data have suggested that nedaplatin is as effective as cisplatin with less digestive and renal toxicity and can be a promising alternative choice for cervical cancer patients [29]. However, larger randomized clinical trials are required to validate this finding.

It is well known that DNA repair plays an important role in platinum resistance and any perturbation in DNA repair may lead to an altered sensitivity to the treatment [30], [31]. DSBs, the most lethal form of DNA damage induced by ionizing radiation and platinum agents, are repaired by two major repair pathways–HRR and non-homologous end-joining [32]. It has been suggested that an elevated frequency of DSBs and chromosomal aberrations are observed 16–24 h after cisplatin exposure [33] and suppression of related proteins involved in the HRR pathway may be responsible for resistance to cisplatin [34]. In *Saccharomyces cerevisiae*, yeast *RAD52* plays a key role in replication-associated HRR [35]. Yeast RAD52 and mammalian RAD52 show similar amino acid sequence and biochemical activities, which suggests its conserved function [36]. Previous data have shown that mammalian RAD52 could respond to DNA DSBs and replication stalling independently of BRCA2, acting as an independent and alternative HRR pathway [37]. For example, the absence of the RAD52 protein resulted in extensive chromosome aberrations, especially chromatid-type aberrations [37], and platinum agents might bind directly to DNA, leading to different types of DNA lesions [38]. Additionally, the downregulation of *RAD52* mRNAs was associated with poor response of cells to platinum-derived compounds in BRCA1-defective HCC1937 cells [15]. Consistently, in the present study, we found that the RAD52-negative protein expression status was associated with poor response of cervical cancer cells to carboplatin. Therefore, *RAD52* may be involved in the formation of platinum resistance. However, additional studies are needed to explore mechanisms underlying the observed low RAD52 expression levels that will be instrumental to cervical cancer chemotherapy.

To the best of our knowledge, this is the first study that focused on the predictive value of *RAD52* for variations in platinum resistance and prognosis in CSCC. Interestingly, we observed that the rs11571378 genotype distribution among the patients departed from HWE but did not predict any of clinical outcomes. In contrast, the other two SNPs (i.e., rs1051669 and rs10774474), whose genotype distributions among the patients followed HWE, were independently associated with the *in vitro* inhibition rates of carboplatin and nedaplatin in CSCC cells, respectively, indicating an important role of *RAD52* variants in platinum resistance. Actually, these two SNPs are both predicted as potentially functional. For example, the rs1051669 SNP is located in the 3′- UTR of *RAD52* and may be at a miRNA binding site disrupt the miRNA–mRNA interaction and affect the expression of miRNA targeted genes [39]. Likewise, the rs10774474 SNP is located in the upstream of *RAD52*, which might be a transcription factor binding site to participate in gene regulatory networks, such as DNA repair pathways [40]. Furthermore, RAD52 protein expression levels were associated with inhibition rates of carboplatin, a similar result observed for the *RAD52*-rs1051669 SNP. Interestingly, our genotype-phenotype correlation analyses also demonstrated that the rs1051669 SNP was significantly associated with RAD52 protein expression levels. Therefore, it seems biologically plausible that the *RAD52*-rs1051669 SNP may be functional by regulating RAD52 expression and thus contribute to platinum resistance in cervical cancer patients.

*Kaplan-Meier* survival estimates further showed that patients who carried at least one (≥1) variant allele of three *RAD52* SNPs had a significantly poorer PFS than those who did not carry any of the variant alleles, indicating the effect of *RAD52* SNPs on the prognosis of CSCC patients. Because these variant genotypes may predict the protein expression, we hypothesized that RAD52 protein expression levels may predict survival in cervical cancer. However, in the present study, we did not observe any associations between single SNPs or RAD52 protein expression levels and survival, which might be due to the relatively short follow-up time with limited events of recurrences and deaths. However, some published data on other cancers do support our hypothesis, although no studies on cervical cancer have been published to date. Recently, Jewell et al. reported that the overexpression of *RAD52* mRNA could predict poor PFS with a hazard ratio of 4.49 in melanoma and that cancer cells with upregulated genes of DNA repair pathways were likely to be more aggressive [41].

In summary, *RAD52* SNPs, either individually or collectively, may modify gene function and alter RAD52 protein expression levels, thus rendering cancer cell resistant to platinum agents. Larger prospective studies with longer follow-up time are required to validate our findings.
